# Effect of Short-Term Treatment with Continuous Positive Airway Pressure on Cardiopulmonary Exercise Tolerance, Pulmonary and Cardiac Function in Patients with Obstructive Sleep Apnea

**DOI:** 10.3390/medicina59020326

**Published:** 2023-02-09

**Authors:** Laima Kondratavičienė, Lina Padervinskienė, Tomas Lapinskas, Eglė Ereminienė, Kęstutis Malakauskas, Marius Žemaitis, Skaidrius Miliauskas

**Affiliations:** 1 Department of Pulmonology, Lithuanian University of Health Sciences, 44307 Kaunas, Lithuania; 2 Department of Radiology, Lithuanian University of Health Sciences, 44307 Kaunas, Lithuania; 3 Department of Cardiology, Lithuanian University of Health Sciences, 44307 Kaunas, Lithuania

**Keywords:** cardiopulmonary exercise testing, lung function tests, cardiac magnetic resonance imaging, obstructive sleep apnea, polysomnography, continuous positive airway pressure

## Abstract

*Background:* Obstructive sleep apnea (OSA) is a condition with a high prevalence, linked to an increased risk of cardiovascular disease as well as increased morbidity and death. CPAP is currently considered the “gold standard” treatment for OSA, but more thorough research and testing are required to assess its efficacy on cardiopulmonary function. *Objectives:* To evaluate pulmonary function of OSA patients, cardiopulmonary exercise tolerance test (CPET) performance, cardiac magnetic resonance imaging (MRI) parameters, and polysomnographic changes before and after 3 months of CPAP therapy. *Materials and methods:* A total of 34 patients diagnosed with moderate or severe OSA, as well as 17 patients as a control group for the evaluation of the cardiac MRI, were included in this study. All the subjects were obese (body mass index (BMI) > 30 kg/m^2^). Lung function tests, CPETs, cardiac MRIs, and polysomnography were performed at the time of the study’s enrolment before the initiation of the CPAP therapy and after 3 months of the CPAP treatment. *Results:* The patients‘ VO_2max_ during the CPAP treatment tended to increase, but no statistical significance was found (before treatment it was 17.52 ± 3.79 mL/kg/min and after 3 months of treatment, it was 18.6 ± 3,4 mL/kg/min; *p* = 0.255). The CPAP treatment had positive effects on pulmonary ventilation at the anaerobic threshold (VE_AT_): 44.51 L/min (43.21%) during the baseline visit and 38.60 L/min (37.86%) after the 3-month treatment period (*p* = 0.028). The ventilator equivalent for the carbon dioxide slope (VE/VCO_2_) at peak exercise decreased from 23.47 to 20.63 (*p* = 0.042). The patients’ pulmonary function tests were without abnormalities and did not change after treatment. When assessing cardiac the MRIs, the RV ejection fraction was lower in the OSA group compared to that of the control subjects (53.69 ± 8.91 and 61.35 ± 9.08, *p* = 0.016). Both LA and RA global longitudinal strains (GLS) improved after 3 months of treatment with CPAP (20.45 ± 7.25 and 26.05 ± 14.00, *p* = 0.043; 21.04 ± 7.14 and 26.18 ± 7.17, *p* = 0.049, respectively). Additionally, it was found that CPAP therapy led to statistical improvements in RV end-diastolic volume (164.82 ± 32.57 and 180.16 ± 39.09, *p* = 0.042). The AHI and oxygen desaturation index (ODI) significantly changed after 3 months of the initiation of the CPAP treatment (*p* = 0.049 and *p* = 0.001, respectively). The REM sleep duration decreased, while the duration of non-REM sleep increased after treatment initiation with CPAP (*p* = 0.016 and *p* = 0.017, respectively). *Conclusions:* Short-term CPAP treatment improves pulmonary ventilation, sleep efficiency, and sleep architecture. Significant alterations in both atrias’ GLS and RV end-diastolic volume were observed after 3 months of treatment. Longer-term follow-up and a larger patient sample are needed to confirm the reproducibility of our results.

## 1. Introduction

Obstructive sleep apnea (OSA) is a chronic disorder characterized by the episodic narrowing of the upper airway during sleep, followed by episodic hypoxia and sleep fragmentation [1,2]. The prevalence of OSA is increasing due to the rise in obesity [2]. Obesity leads to the relative narrowing of airway lumen and increases the likelihood of obstructions [3]. OSA, along with obesity, is a significant risk factor for serious cardiovascular diseases, including arterial hypertension, ischemic heart disease, heart failure, and stroke [3,4]. It is associated with the remodeling of the left heart. Most studies assessed changes in the heart by using echocardiography. Our previous study proved that a short-term continuous positive airway pressure (CPAP) treatment improved the function of the left ventricle [5]. Cardiac magnetic resonance imaging (MRI) is one of the best diagnostic tools for the quantitative assessment of the size and functional parameters of the right and left ventricles due to three-dimensional volume imaging [6]. Traditional cardiac MRIs have a significant impact in determining atrias’ volume, but only a minor role in determining their function. The feasibility and reproducibility of advanced cardiac MRI feature tracking (FT) as a method for determining atrias’ function through strain are becoming more and more evident [7]. Another diagnostic tool for assessing exercise tolerance by measuring cardiovascular and ventilatory functions is the cardiopulmonary exercise test (CPET). The CPET provides information about the cardiopulmonary and oxygen transport systems, muscles, and metabolic activity of tissues that interact during exercise [8]. Polysomnography (PSG) in the sleep laboratory is still the one and only test used for the diagnosis of OSA, and adjusting patients’ CPAP is the gold standard of treatment.

Recent studies have found that due to increased energy demands, patients with OSA are frequently more physically exhausted during exercise, have weaker muscles, and have poorer physical reserves [9,10]. CPETs, as a non-invasive technique, provide a quantitative, objective assessment of metabolic, pulmonary, and cardiovascular responses to exercise [11]. There is disagreement over how CPAP therapy affects the CPET-measured exercise tolerance of individuals with OSA. Quadri et al. aimed to evaluate the impact of CPAP on exercise performance and cardiovascular autonomic anomalies in patients with OSA who did not alter their lifestyle or weight while receiving therapy [12]. The main finding was that 2 months of CPAP treatment improved oxygen consumption (VO_2_) and physical exercise tolerance, although their BMIs did not change. Another study by Lin with co-authors also aimed to examine effect of nasal CPAP on CPET test performance throughout the same period of time [13]. They found out that during this short period, CPAP treatments improved VO_2_, workload, anaerobic threshold, and oxygen pulse. However, Fernandez et al. study revealed no noticeable difference in oxygen VO_2_ throughout the course of a 3-month therapy period [14]. 

The duration of CPAP treatments varied greatly in the studies (from a few days to several weeks), making it difficult to distinguish between short- and long-term effects. Additionally, OSA patients with a wide range of other health issues were evaluated, adding to the controversy related to this subject.

The goal of study was to compare the changes in lung function, CPET performance, cardiac MRI parameters, and polysomnography in OSA patients before and after 3 months of CPAP therapy.

## 2. Material and Methods

### 2.1. Subjects

This study was a part of ongoing research. Patients’ quality of life was assessed in Section 1 of this research study (using the same clinical trial participants), and the echocardiographic and serum biomarker changes following CPAP treatment in OSA patients were assessed in Section 2 [5,15].

Thirty-four people with moderate to severe OSA were enrolled in the research study from January 2020 to June 2021. Seventeen patients underwent the final investigation 3 months after beginning CPAP therapy. Patients who stopped participating in the study either lost contact in follow-up or were denied CPAP therapy. Only the patients in this trial who had completed CPAP treatment for three months were examined.

The Kaunas Regional Biomedical Research Ethics Committee approved the project (no. BE-2-23, 19 May 2020, Kaunas, Lithuania).

Being between 18 and 65 years old, having a diagnosis of moderate to severe OSA as well as a body mass index (BMI) of at least 30 kg/m^2^, and signing an informed permission form were the inclusion criteria.

Exclusion criteria were the following: subjects under the age of 18 and adults above the age of 65; and subjects with clinically significant ischemic heart disease, severe valvular heart disease (grade 3), or uncontrolled arterial hypertension (AH). The absence of a completed informed consent form and the investigator’s discretion were other exclusion criteria.

All patients underwent comprehensive clinical investigations, which included in-depth physical examinations and the recording of their symptoms, medical and surgical histories, and comorbidities. In order to rule out any nasal disease (such as nasal polyposis, a deviated nasal septum, or an insufficiency of the nasal valve), patients were checked and seen by an otorhinolaryngologist at the Hospital of Lithuanian University of Health Sciences (LUHS) Kaunas Clinics Outpatient Clinic.

People suspected of having OSA were referred to the sleep laboratory at the LUHS Kaunas Clinics Pulmonology Department for an overnight diagnostic PSG. Using the Alice 6 LDx diagnostic sleep machine, PSG was performed (Philips Respironics, Murrysville, PA, USA). Hypopnea was defined as a decrease in airflow for at least 10 s that was also followed by a 3% drop in SpO2 or arousal. Apnea was defined as the lack of airflow for longer than 10 s. The apnea–hypopnea index (AHI) was calculated throughout the trial for each hour of sleep. Patients with OSA were categorized into three groups based on their AHI scores: those with mild OSA (AHI 5 but 15), moderate OSA (AHI 15 but 30), and severe OSA (AHI 30) [16,17].

Following the diagnosis of OSA, each patient spent an extra night in the sleep laboratory to undergo manual CPAP titration, which establishes the optimal pressure at which the CPAP machine stops causing abnormal breathing events. Patients were recruited to participate in the clinical study if they had moderate or severe OSA.

The control group was chosen for comparison of cardiac MRI parameters only and consisted of 17 healthy, non-obese (BMI < 30 kg/m^2^) subjects without any concomitant diseases. The control group underwent cardiac MRIs because there was a suspicion of myocardial pathology. However, the results of the examinations showed no evidence of myocardial injury, including good general systolic function in both ventricles, good regional contraction, unchanged valvular anatomy and function, and no evidence of myocardial fibrosis in the late gadolinium enhancement sequence.

### 2.2. Pulmonary Function Tests

#### 2.2.1. Spirometry

Lung function was assessed by measuring forced vital capacity (FVC), forced expiratory volume in 1 s (FEV_1_), and the forced expiratory volume in 1 s to vital capacity ratio (FEV_1_/VC) using a Ganshorn spirometer (Ganshorn Medizin Electronic, Niederlauer, Germany), and by comparing the values obtained with the required size calculated according to the standard methodology based on age, height, and sex. For each measurement, the highest value of the measured indicator was selected for analysis after determining that the blow-out was technically correct.

#### 2.2.2. Lung Diffusion Test

A single inhalation method was used for gas diffusion (Ganshorn Medizin Electronic, Niederlauer, Germany). The subject maximally inhaled (to total lung capacity (TLC)) a gas mixture with a low carbon monoxide (CO) concentration (~0.3 percent) and held their breath for 10 s. The CO concentration of the exhaled alveolar air was examined.

The diffusing capacity of the lung for CO (DLCO) was calculated, which indicates how much CO diffuses from the alveolar air into the pulmonary capillary blood in one minute at a pressure gradient of 1 kPa. The DLCO was adjusted according to the subject’s hemoglobin concentration.

#### 2.2.3. Total Body Plethysmography

Total body plethysmography was performed in a special hermetic plethysmography chamber, Ganshorn Power Cube Body+ (Ganshorn Medizin Electronic, Niederlauer, Germany). At the end of exhalation, the valve closed the airflow in the mouthpiece and the subject inhaled and exhaled without effort. Lung volumes were measured according to the principle of the plethysmographic chamber pressure difference. The following indicators were evaluated in this study: specific airway conductance (sGaw), specific airway resistance (sRaw), functional residual capacity (FRC), residual volume (RV), total lung capacity (TLC), and residual volume–total lung capacity ratio (RV/TLC) (mean values of four correctly performed maneuvers). Ventilatory data were interpreted according to international recommendations [18].

### 2.3. Cardiopulmonary Exercise Testing

Each patient was subsequently evaluated with incremental, maximal, ECG-monitored CPETs (Schiller CS-200, Munich, Germany). All tests were performed using an ergospirometer (Schiller CS-200, Munich, Germany). The criteria for exhaustion were a Borg rating associated with a respiratory exchange ratio (RER) > 1.10, a peak heart rate (HR) ≥ 90% of the predicted HR max, and/or the achievement of a plateau in oxygen uptake (VO_2_) [13]. Arterial blood pressure and peripheral oxygen saturation were continuously monitored. Measurements of ventilatory and cardiovascular limitation were sampled breath-by-breath to assess O_2_ uptake/consumption (VO_2_), CO_2_ production (VCO_2_), minute ventilation (VE), ventilatory equivalents for carbon dioxide (VE/VCO_2_) slope at peak exercise, and O_2_ pulse. VE, VO_2_, and VCO_2_ were also determined at the anaerobic threshold (AT) and respiratory compensation point (RCP) [19].

### 2.4. Cardiac Magnetic Resonance Imaging

Cardiac MRIs were performed according to the approved protocol of the Hospital of the Lithuanian University of Health Sciences [20,21]. They were performed with a 1.5 T whole-body system (Siemens Aera, Siemens Medical Solutions; Erlangen, Germany) at baseline and after 3 months (follow-up visit). Image analysis was performed using a commercial software package (Medis Medical Imaging Systems bv, Leiden, The Netherlands). A breath-hold balanced steady-state free precession sequence was used to generate bright-blood cine images in the four-chamber long-axis and two-chamber long-axis planes. Left ventricle (LV) mass was calculated by summing the slices, manually tracing the endocardial and epicardial contours in each end-diastolic slice, and then multiplying by the slice thickness to obtain the myocardial volume. Both ventricles’ end-diastolic and end-systolic volumes (EDV and ESV) were measured. Ventricle stroke volume (SV) and ejection fraction (EF) were calculated from these volumes. Both ventricles’ EDV, ESV, and SV were indexed for body surface area.

The Medis Suite Q Strain 2.0 software program was used to semi-automatically delineate the endocardial surfaces during the cardiac cycle and perform feature tracking (FT) analysis. The outlines were examined, and if necessary, manually modified. By averaging the strain curves from the two-chamber, three-chamber, and four-chamber long-axis views, the LV global longitudinal strain (GLS) was determined. By averaging the strain curves of the basal, mid, and apical segments derived from the short-axis images, the LV global circumferential strain (GCS) was determined. In the cardiac four-chamber long-axis perspective, RV GLSs were computed. By averaging the strain curves of the four-chamber and two-chamber long-axis images, the right atrium (RA) and left atrium (LA) GLSs were determined [21].

### 2.5. Interventions and Follow-Up

All study subjects were asked to return after three months of starting treatment for a follow-up. Data from the CPAP machine were downloaded to determine adherence to CPAP treatment. We defined treatment adherence as using the CPAP machine for more than 4 h per night on more than 70% of nights and having an AHI < 5. Lung function tests, CPETs, and cardiac MRIs were performed after three months of treatment with CPAP.

### 2.6. Statistical Analysis

We analyzed the data of patients being treated with CPAP (OSA group; values at baseline and 3 months after the start of CPAP treatment). Quantitative variables are described as the mean and standard deviation, or the median and 25th/75th percentiles. To describe the distribution and changes in all quantitative variables, the nonparametric Mann–Whitney U test for two dependent samples was used.

IBM SPSS Statistics for Windows, version 27.0 (IBM Corp., Armonk, NY, USA), was used for statistical analysis. Statistical significance was considered to be *p* < 0.05.

## 3. Results

### 3.1. Baseline Data Characteristics and Sleep Measurements

Seventeen patients with newly diagnosed moderate or severe OSA without significant comorbidities that could affect the study results participated. Fifteen males and two females with a mean age of 52.15 ± 8.89 were included in this study. The male gender was more prevalent in our study group. The included participants had a mean baseline BMI of 39.23 ± 6.1 kg/m^2^, and a mean AHI of 76.14 ± 24.32 events/hour. BMI did not change during the 3-month CPAP treatment period (*p* > 0.05) (Table 1). Concomitant disorders included well-controlled arterial hypertension in 14 (82%) patients, type 2 diabetes in 7 (50%) patients, gout in 4 (24%) patients, rheumatoid arthritis in 1 (6%) patient, and well-controlled asthma in 1 (6%) patient.

The patients’ AHI and oxygen desaturation index (ODI) significantly decreased 3 months after the CPAP treatments, compared to the baseline measurements (*p* = 0.049 and *p* = 0.001, respectively). Their sleep architecture, characterized by REM and non-REM sleep stages, changed in the 3-month CPAP treatment period. Their REM sleep duration decreased, while the duration of non-REM sleep increased after the treatment initiation of CPAP (*p* = 0.016 and *p* = 0.017, respectively). The other polysomnographic data before and after the 3 months of CPAP treatment did not change (Table 1).

### 3.2. CPETs and Pulmonary Function Tests

The results of the CPET data are shown in Table 2. The main VO_2max_ prior to the CPAP treatment was 84.36 ± 18.08 (VO_2max_ > 80% is regarded as the normal value), and in absolute values, it was 17.52 ± 3.79 mL/kg/min. The VO_2max_ during the CPAP treatment tended to increase, but no statistical significance was found (after 3 months of treatment, it was 82.21%—18.6 mL/kg/min; *p* = 0.255). The CPAP treatment also had positive effects on work performance (maximum load, 79.86% to 85.5%; *p* = 0.093). The pulmonary ventilation at the anaerobic threshold (VE_AT_) during the baseline visit was 44.51 L/min (43.21%) and significantly decreased after the 3-month treatment period (38.60 L/min—37.86%; *p* = 0.03 and *p* = 0.028, respectively). The ventilator equivalent for carbon dioxide slope (VE/VCO_2_) at peak exercise decreased from 23.47 to 20.63 during the short-term CPAP treatment period (*p* = 0.042).

After the 3-month CPAP therapy period, the patients’ pulmonary function tests were normal and did not change (Table 3).

### 3.3. Cardiac MRI Data

When comparing the baseline cardiac MRI parameters of the OSA group with those of the control group, several statistically significant parameters were found. The MRI parameters of the left heart did not differ. The RV end-diastolic volume was higher in the OSA group compared to that of the control group (70.46 ± 14.77 mL/m^2^ and 58.65 ± 14.31 mL/m^2^, *p* = 0.042). It was also found that the RV ejection fraction was lower in the OSA group compared with that of the control subjects (53.69 ± 8.91% and 61.35 ± 9.08%, *p* = 0.016) (Table 4).

Both the LA and RA global longitudinal strains improved (GLS) after 3 months of treatment with CPAP (20.45 ± 7.25% and 26.05 ± 14.00%, *p* = 0.043; 21.04 ± 7.14 and 26.18 ± 7.17, *p* = 0.049, respectively). Although there was a trend toward improvement, the RA GLS did not change considerably in the meantime (−24.21 ± 14.32% and −27.31 ± 5.19%, *p* = 0.642). Additionally, it was discovered that CPAP therapy led to statistical improvements in RV end-diastolic volume (164.82 ± 32.57 mL and 180.16 ± 39.09 mL, *p* = 0.042), although the RV EF did not significantly change (Table 5).

## 4. Discussion

This study examined the effects of short-term CPAP therapy on exercise tolerance and pulmonary and cardiac function in newly diagnosed obese patients with moderate to severe OSA who did not have any comorbid illnesses. The main findings of this study were that CPAP treatment improved patients’ exercise capacity (a tendency has been detected, even if it is not statistically significant) and pulmonary ventilation measured by CPETs and the function of both atria on cardiac MRIs.

We found that CPAP treatment reduced the AHI and ODI while improving sleep efficiency and sleep architecture. There were no drug changes that could have affected sleep architecture.The fact that the patients slept in an unfamiliar bed for the research on sleep could have prevented them from getting a deep night’s sleep. Lack of sleep can affect how well the body responds to carbon dioxide and hypoxia. Sleep, on the other hand, restores cellular activity and cellular function, particularly in the brain and in the muscles that raise VO_2max_ during exercise. The enhanced daytime alertness after treatment with CPAP may also help with patients‘ motivation and CPET performance, with an accompanying rise in VO_2max_ [22,23].

The results from the CPETs in the OSA patients who received CPAP are inconsistent and controversial. Patients’ VO_2_ and workload before the initiation of treatment are lower, showing an exercise limitation. Age, BMI, and exercise habits are just a few of the many variables that have been suggested to potentially affect CPET results. Age and BMI did not significantly differ in our study either before or after the CPAP therapy. During the study period, neither the patients’ exercise routines nor the exercise instruction they received changed. Therefore, rather than being due to other complicating factors, it is likely that the benefits we saw were due to the CPAP therapy. We observed that workload and VO_2_ tended to increase, although no statistical significance was found. Ozsarac et al. investigated a 4-week treatment with CPAP response [24]. In their study, a significant decrease in the ventilator equivalent for carbon dioxide (VE/VCO_2_) was observed even after 4 weeks of the treatment, which was also found in our study, only with a longer treatment period. Hargens et al. evaluated ventilatory responses during CPETs in obese healthy controls and obese OSA patients [25]. The main finding was that the VO_2max_ was similar, but the VE, VE/VCO_2_, and VE/VO_2_ were increased in OSA patients compared with the controls using a cycle ergometer. They explained that this effect was due to repeated episodes of hypoxia, which cause an increased ventilatory response to exercise. Myers et al. demonstrated that the VE/VCO_2_ slope and VE/VCO_2_ at the AT can be good prognostic predictors of chronic lung and heart disease [26]. The changes in VE/VCO_2_ at the AT suggest that CPAP therapy reverses the pathophysiological changes in OSA, which may improve cardiac function and allow more efficient ventilation.

The study by Mortari et al. also examined the effects of CPAP treatment on physical performance over a 3-month period [27]. A significant improvement in cardiovascular parameters (heart rate association with BMI and blood pressure) was observed at rest and during submaximal exercise. The downregulation of sympathetic activity during sleep could be the main reason for this cardiovascular response to CPAP [28]. The results of clinical studies on exercise capacity are controversial. In our study, the VO_2_ did not change significantly, but it had a tendency to increase. Various previous studies found increased peak VO_2_ during incremental exercise testing over 2–8-month CPAP treatment periods [29,30]. However, Fernandez et al. study showed no significant improvement in VO_2_ over a 3-month treatment period [14]. These discrepancies may be due to differences in treatment adherence, disease severity, or physical activity levels. Therefore, it appears that CPAP therapy for several weeks is sufficient to reduce the noxious pathophysiologic factors associated with OSA and to improve physical performance (or to show a tendency to improve).

We found no significant changes in the patients’ lung volumes in spirometry, lung diffusion tests, and total body plethysmography. Spirometry abnormalities have been associated with OSA and may occur as these patients have various functional and structural upper airway abnormalities during sleep [31]. Kunos et al. found significant FEV_1_ changes in OSA patients compared to a control group [32]. However, this finding is controversial, as no information about the concomitant diseases or smoking habits of these subjects was reported. Pigakis and co-authors also found a significant increase in the FEV_1_ value after 3 months of CPAP treatment [33], but they found no reason for this change. In addition, this might be due to obesity, as it leads to restrictive ventilatory defects. The tendency for bronchodilation in OSA patients may be due to the overnight increase in blood epinephrine levels, as demonstrated in Baruzi et al. study [34]. The mechanism of lung volume changes could be influenced by obesity. Obesity can lead to the development of airway hyper-responsiveness [35]. The underlying mechanisms are due to increased abdominal and chest wall masses, shallow breathing, and circulating inflammatory cytokines and hormones (such as leptin and adiponectin) [35]. However, apart from obesity, we could not find any other explanation or reason for our results, as no change in lung function during treatment with CPAP was observed.

One of the first studies to investigate the effect of short-term (3-month) CPAP treatment using cardiac MRIs was conducted by Magalang UJ et al. [36]. The main finding was a decrease in RV volumes. According to our data, we observed a tendency for the RV volume to change. Echocardiographic data on the RV are limited. Compared witch echocardiography, morphologic assessments using MRIs are more accurate. Our findings show that both atrias’ GLS improved, which was not the result in our previous echocardiographic investigation [5]. A cardiac MRI FT analysis provides a higher spatial resolution, superior contrast, and greater reproducibility when used under the same conditions. By demonstrating deficits prior to the development of an atrial enlargement, a thorough assessment of the three phases of atrial function using this technique can assist physicians in making decisions and enhance patient outcomes [7].

An interesting study by Xu L. and co-authors [37] was published in the Journal of the American Heart Association. They compared obese and non-obese OSA patients with non-obese healthy subjects by performing cardiac MRIs and assessed the CPAP treatment effect over a 4-month follow-up period. It was found that the LV end-diastolic volume (LV EDV) was significantly higher in the OSA group compared with that of the subjects without OSA. Our study did not include non-obese patients with OSA. According to our data, no statistical significance of the LV EDV changes was observed in the groups, but a tendency for higher parameters was demonstrated in the OSA group. One more finding in this study was that CPAP treatment significantly changed the LA volume index. In various studies, a high LA volume measured by MRI has been shown to be an independent factor of clinical cardiovascular events [38]. These data demonstrate that a short period of CPAP treatment (3 months in our study and 4 months in the study of Xu L.) is too short to assess the effect of cardiac MRI parameters.

The long-term CPAP treatment effect was also investigated by Wuest et al. [6]. Their study assessed changes in cardiac MRI parameters after 7 months of CPAP treatment. CPAP therapy improved the LV stroke volume (LV SV) and RV ejection fraction (RV EF) in OSA patients. All other parameters did not change. However, one of the main conclusions of this study was that these results need to be confirmed in a larger patient population.

## 5. Limitations of This Study

It is essential to emphasize the limitations of this clinical study. The relatively small patient sample is the primary limitation of this study. Another limitation might be that obese OSA patients were not compared with obese patients without an OSA diagnosis, healthy controls, or patients with cardiac pathology. The tests that were performed are thought to show more major changes after a longer follow-up period.

## 6. Conclusions

Short-term CPAP treatment tends to improve exercise capacity and pulmonary ventilation, but further studies with larger subject groups are needed. CPETs could be a tool for compliance with CPAP treatments and may be used in clinical practice. Although CPAP therapy had no effect on the lung volumes as measured by pulmonary function tests, they may improve with changes in BMI. Cardiac MRIs can produce high-quality images for a precise and comprehensive noninvasive assessment of heart anatomy and function, as well as to assess subclinical changes in the right heart. As determined by our cardiac MRI FT analysis, CPAP therapy improves both atrias’ function. A longer-term follow-up and comparison with individuals with OSA who have more severe cardiac dysfunction and healthy controls may provide new insights into the link between OSA and cardiovascular disease.

## Figures and Tables

**Table 1:** Baseline characteristics before and after CPAP.

		Before Treatment (*n* = 17)	3 Months after CPAP Treatment (*n* = 17)	*p*-Value *
		Mean (Standard Deviation)		
	BMI, kg/m^2^	39.23 (6.1)	38.3 (5.5)	*p* > 0.05
Polysomnography	AHI, per h	76.14 (24.32)	67.73 (26.64)	0.049 *
	ODI	71.42 (23.95)	29.24 (31.05)	0.001 *
	Mean SpO_2_ (%)	91.53 (2.26)	92.56 (2.19)	0.144
	Minimum SpO_2_ (%)	66.42 (12.84)	72.00 (11.21)	0.099
	TST (min)	361.74 (61.25)	348.54 (55.44)	0.356
	Non-REM (%)	80.14 (6.53)	85.25 (5.21)	0.017 *
	REM (%)	19.96 (7.37)	14.75 (7.45)	0.016 *
	Sleep efficiency (%)	76.93 (12.61)	82.05 (12.01)	0.037 *
	Arousal index, per h	75.82 (23.39)	72.28 (23.91)	0.309

* *p*-value < 0.05, according to the nonparametric Wilcoxon test. CPAP: continuous positive airway pressure; AHI: apnea–hypopnea index; BMI: body mass index; ODI: oxygen desaturation index; TST: total sleep time; REM: rapid eye movement.

**Table 2:** CPET data.

	Before Treatment (*n* = 17)	3 Months after CPAP Treatment (*n* = 17)	*p*-Value *
Maximum load (%)	79.86 (15.8)	85.5 (16.35)	0.093
Maximum load (W)	170.36 (39.25)	174.29 (45.18)	0.662
VO_2_ (max. load) (L/min)	2.14 (0.41)	2.23 (0.44)	0.706
VO_2_ (max. load) (%)	84.36 (18.08)	87.21 (17.48)	0.706
VO_2_ (max. load) (mL/kg/min)	17.52 (3.79)	18.6 (3.40)	0.255
VO2/kg (max. load) (%)	83.77 (18.68)	88.92 (18.36)	0.195
VCO_2_ (max. load) (L)	2.61 (0.61)	2.67 (0.67)	0.889
VCO_2_ (max. load) (%)	93 (20.21)	93.86 (18.63)	1
HR rest (beats/min)	86.08 (10.10)	88.83 (12.47)	0.906
HR max (beats/min)	135.5 (20.66)	132.38 (18.13)	0.432
HR max (%)	90.25 (12.74)	88 (12.14)	0.283
O_2_ pulse (max. load) (mL/beat)	16.69 (3.11)	16.46 (3.66)	0.925
O_2_ pulse (max. load) (%)	72.64 (16.57)	72.14 (16.53)	0.826
VE_AT_ (L/min)	44.51 (10.59)	38.60 (7.52)	0.03 *
VE_AT_ (%) (reference value)	43.21 (21.19)	37.86 (17.77)	0.028 *
VE max load	74.53 (20.04)	69.81 (17.77)	0.51
VE max load (%)	68.84 (22.18)	66.36 (24.24)	0.615
VE/VCO_2_ slope (peak value)	23.47 (2.73)	20.63 (3.52)	0.042 *
RER max. load	1.19 (0.14)	1.21 (0.09)	0.109

* *p*-value < 0.05, according to the nonparametric Wilcoxon test. Data values are presented at maximum load and anaerobic threshold. VO_2_: O_2_ consumption; VCO_2_: CO_2_ production; AT: anaerobic threshold; HR: heart rate; O_2_ pulse: oxygen pulse; VE: pulmonary ventilation; VE at AT: pulmonary ventilation at the anaerobic threshold; VE/VCO_2_: ventilator equivalent for carbon dioxide; RER: respiratory exchange ratio; max.: maximum.

**Table 3:** Lung function test (spirometry, lung diffusion test, total body plethysmography) data.

	Before Treatment (*n* = 17)	3 Months after CPAP Treatment (*n* = 17)		*p*-Value *
	Mean (Standard Deviation)			
Spirometry	FVC (l)	4.43 (0.95)	4.38 (0.72)	0.666
	FVC (%)	90.31 (11.84)	89.64 (8.29)	0.906
	FEV_1_ (l)	3.59 (0.78)	3.54 (0.59)	0.906
	FEV_1_ (%)	93.46 (13.79)	92.57 (9.44)	0.969
	FEV/VC	81.12 (3.31)	81.17 (3.78)	0.221
	FEV_1_/VC (%)	102.92 (4.27)	102.86 (4.46)	0.404
Lung diffusion test	DLCO (%)	92.94 (8.57)	93.84 (8.35)	0.683
Total body plethysmography	sGAW	0.87 (0.27)	1.00 (0.24)	0.099
	sGAW (%)	99.67 (30.39)	114.78 (0.43)	0.099
	sRAW	1.24 (0.43)	1.05 (0.27)	0.096
	sRAW (%)	109.0 (38.41)	92 (22.08)	0.109
	FRC	2.94 (0.58)	3.06 (0.52)	0.490
	FRC (%)	84.21 (15.1)	87.21 (11.53)	0.593
	RV	2.21 (0.36)	2.31 (0.47)	0.49
	RV (%)	99.43 (13.23)	102.71 (16.63)	0.638
	TLC	6.49 (0.96)	6.72 (0.96)	0.149
	TLC (%)	91.78 (9.98)	95.07 (9.15)	0.116
	RV/TLC	34.33 (5.06)	34.64 (7.04)	1.0
	RV/TLC (%)	99.21 (10.49)	99.00 (14.52)	0.925

* *p*-value < 0.05, according to the nonparametric Wilcoxon test. FVC: forced vital capacity; FEV_1_: forced expiratory volume in 1 s; FEV_1_/VC: the ratio of the forced expiratory volume in the first one second to the forced vital capacity of the lungs; DLCO: diffusing capacity of carbon monoxide; sGAW: specific airway conductance; sRAW: specific airway resistance; FRC: functional residual capacity; RV: residual volume; TLC: total lung capacity; RV/TLC: the ratio of the residual volume to the total lung capacity.

**Table 4:** Cardiac MRI parameters of control and OSA group patients.

	OSA (*n* = 32)	Control (*n* = 17)	*p*-Value *
	Mean (Standard Deviation)		
LV parameters			
LV EDVI (mL/m^2^)	79.44 (21.02)	71.58 (15.59)	0.438
LV ESVI (mL/m^2^)	30.89 (11.69)	27.47 (10.94)	0.438
LV EF (%)	61.62 (5.28)	62.71 (9.45)	0.506
LV GLS (%)	−24.86 (4.85)	−23.64 (4.89)	0.532
LV GCS (%)	−35.14 (4.67)	−39.11 (7.01)	0.063
RV parameters			
RV GLS (%)	−25.72 (10.93)	−21.54 (5.87)	0.096
RV EDVI (mL/m^2^)	70.46 (14.77)	58.65 (14.31)	0.042 *
RV ESVI	33.56 (11.67)	22.88 (9.19)	0.069
RV EF (%)	53.69 (8.91)	61.35 (9.08)	0.016 *

* *p*-value < 0.05, according to the nonparametric Wilcoxon test. OSA: obstructive sleep apnea; LV: left ventricle; RV: right ventricle; LA: left atrium; RA: right atrium; EDVI: end-diastolic volume index; ESVI: end-systolic volume index; EF: ejection fraction; GLS: global longitudinal strain; GCS: global circumferential strain.

**Table 5:** Changes in cardiac MRI parameters in OSA group patients after treatment with CPAP.

	Before Treatment (*n* = 17)	3 Months after CPAP Treatment (*n* = 17)	*p*-Value *
	Mean (Standard Deviation)		
LA and LV parameters			
LV EDV (mL)	182.68 (43.81)	197.93 (30.28)	0.102
LV EDVI (mL/m^2^)	75.92 (17.51)	81.79 (10.67)	0.136
LV ESV (mL)	70.03 (21.58)	77.66 (16.17)	0.163
LV ESVI (mL/m^2^)	29.12 (8.86)	32.26 (7.04)	0.193
LV SV (mL)	114.43 (26.31)	122.47 (20.14)	0.210
LV SVI (mL/m^2^)	47.49 (10.14)	50.63 (7.26)	0.246
LV EF (%)	61.78 (4.89)	62.08 (5.94)	0.906
LV mass	125.65 (30.39)	120.39 (26.13)	0.266
LA (cm^2^)	31.61 (9.16)	25.65 (5.13)	0.724
LV GLS (%)	−23.76 (4.10)	−24.29 (3.91)	0.981
LV GCS (%)	−35.42 (4.79)	−34.88 (5.51)	0.850
LA GLS (%)	20.45 (7.25)	26.05 (14.00)	0.043 *
RA and RV parameters			
RV EDV (mL)	164.82 (32.57)	180.16 (39.09)	0.042 *
RV EDVI (mL/m^2^)	68.82 (12.74)	74.81 (14.74)	0.067
RV ESV (mL)	78.33 (23.64)	77.45 (21.77)	0.846
RV ESVI (mL/m^2^)	32.53 (9.27)	32.94 (8.21	0.791
RV EF (%)	53.35 (9.36)	57.09 (7.51)	0.151
RA (cm^2^)	21.94 (2.68)	23.48 (4.19)	0.129
RV GLS (%)	−24.21 (7.37)	−27.31 (5.19)	0.480
RA GLS (%)	21.04 (7.14)	26.18 (7.17)	0.043 *

* *p*-value < 0.05, according to the nonparametric Wilcoxon test. OSA: obstructive sleep apnea; LV: left ventricle; RV: right ventricle; LA: left atrium; RA: right atrium; EDVI: end-diastolic volume index; ESVI: end-systolic volume index; SV: stroke volume; SVI: stroke volume index; EF: ejection fraction; GLS: global longitudinal strain; GCS: global circumferential strain.

## Data Availability

Not applicable.
